# Investigating Individuals’ Perceptions Regarding the Context Around the Low Back Pain Experience: Topic Modeling Analysis of Twitter Data

**DOI:** 10.2196/26093

**Published:** 2021-12-23

**Authors:** Robert, Pari Delir Haghighi, Frada Burstein, Donna Urquhart, Flavia Cicuttini

**Affiliations:** 1 Department of Human-Centred Computing Faculty of Information Technology Monash University Caulfield East Australia; 2 Department of Epidemiology and Preventive Medicine School of Public Health and Preventive Medicine Monash University Melbourne Australia

**Keywords:** low back pain, Twitter, content analysis, social media, topic modeling, patient-centered approach, pain experience, context of pain

## Abstract

**Background:**

Low back pain (LBP) remains the leading cause of disability worldwide. A better understanding of the beliefs regarding LBP and impact of LBP on the individual is important in order to improve outcomes. Although personal experiences of LBP have traditionally been explored through qualitative studies, social media allows access to data from a large, heterogonous, and geographically distributed population, which is not possible using traditional qualitative or quantitative methods. As data on social media sites are collected in an unsolicited manner, individuals are more likely to express their views and emotions freely and in an unconstrained manner as compared to traditional data collection methods. Thus, content analysis of social media provides a novel approach to understanding how problems such as LBP are perceived by those who experience it and its impact.

**Objective:**

The objective of this study was to identify contextual variables of the LBP experience from a first-person perspective to provide insights into individuals’ beliefs and perceptions.

**Methods:**

We analyzed 896,867 cleaned tweets about LBP between January 1, 2014, and December 31, 2018. We tested and compared latent Dirichlet allocation (LDA), Dirichlet multinomial mixture (DMM), GPU-DMM, biterm topic model, and nonnegative matrix factorization for identifying topics associated with tweets. A coherence score was determined to identify the best model. Two domain experts independently performed qualitative content analysis of the topics with the strongest coherence score and grouped them into contextual categories. The experts met and reconciled any differences and developed the final labels.

**Results:**

LDA outperformed all other algorithms, resulting in the highest coherence score. The best model was LDA with 60 topics, with a coherence score of 0.562. The 60 topics were grouped into 19 contextual categories. “Emotion and beliefs” had the largest proportion of total tweets (157,563/896,867, 17.6%), followed by “physical activity” (124,251/896,867, 13.85%) and “daily life” (80,730/896,867, 9%), while “food and drink,” “weather,” and “not being understood” had the smallest proportions (11,551/896,867, 1.29%; 10,109/896,867, 1.13%; and 9180/896,867, 1.02%, respectively). Of the 11 topics within “emotion and beliefs,” 113,562/157,563 (72%) had negative sentiment.

**Conclusions:**

The content analysis of tweets in the area of LBP identified common themes that are consistent with findings from conventional qualitative studies but provide a more granular view of individuals’ perspectives related to LBP. This understanding has the potential to assist with developing more effective and personalized models of care to improve outcomes in those with LBP.

## Introduction

Low back pain (LBP) is the leading cause of disability worldwide [1,2]. Approximately 50%-80% of adults experience LBP at least once in their lives [3] and it is a leading cause of work absence and limits physical activities, posing a large economic burden [1,4]. In the United States, the total cost associated with LBP exceeds US $100 billion per year [5,6]. It is also a significant contributor to the current global epidemic of narcotic prescriptions [7].

Optimizing management of conditions such as LBP requires consumers to be engaged in their care. To enable this, health care providers need to have an understanding of the full context of the condition from the consumer perspective. “Contextual variables” here refer to any type of useful information about the context of an individual’s pain experience, such as physical, emotional, social, and/or occupational variables [8]. A better understanding of the contextual variables of individuals with LBP could provide clinicians and health providers with an alternative insight into patients’ concerns, beliefs, and expectations, and has the potential to improve outcomes in LBP [9]. Although there have been many studies examining individuals’ beliefs about LBP, patients’ perspectives remain inadequately understood [10]. Although qualitative studies—including systematic scoping reviews—investigating patients' needs and expectations have been conducted, these have largely focused on a single topic, such as health care, with the findings extrapolated from heterogeneous studies that are of poor quality [11-13]. A further limitation of current approaches is that most traditional data collection methods use predefined frameworks that have the potential to constrain responses. For instance, validated questionnaires that provide statements about back pain and its consequences (such as “back pain must be rested”) and require the respondent to indicate their level of agreement on a scale are commonly used [12,13]. Moreover, for logistical and methodological reasons, many studies restrict the selection of populations to be studied.

With the current advances in online and web technologies, social media has emerged as a new and rich source of first-person health care data [14-16]. Social media platforms provide an opportunity to rapidly collect data from a larger and more diverse population in a cost-efficient manner. Health-related topics are commonly discussed on Twitter [17-19], a microblogging social media site [20]. A systematic review conducted by Sinnenberg et al [21] found six main uses of Twitter in health research: content analysis, surveillance, engagement, recruitment, intervention, and network analysis. Aggregation and analysis of large volumes of health-related data from social media sites could provide valuable information from a first-person point of view [14,22]. In the area of LBP, this approach could be used to investigate individuals’ perspectives and the context around the LBP experience [15,23]. We hypothesize that the detected topics identify specific contexts around the LBP experience in individuals. Thus, the aim of this study was to identify contextual variables of the LBP experience from a first-person perspective using a topic modeling approach of Twitter data to provide useful insights into individuals’ beliefs and perceptions. This has the potential to inform more effective patient-centered approaches to the management of LBP.

## Methods

### Study Approach

Our study approach was to undertake content analysis of Twitter data by applying topic modeling. Content analysis is a widely used technique for qualitative research [24] that enables studying patient experience in depth by deriving topics of interest from text documents [14,25].

### Twitter Data

Twitter was used as the data source rather than other social media platforms, blog posts, or news articles because individuals use this platform for expressing and sharing their feelings and opinions on health-related topics by posting short messages that can be easily collected through application programming interfaces (APIs) or other open sources [14-17,26]. We used an open-source Twitter scraping tool called Twint [27] for collecting tweets related to LBP that were written in English. Twint enables the collecting of Twitter data without using Twitter's API through its publicly available library in the Python programming language [27,28]. We collected tweets posted between January 1, 2014, and December 31, 2018 (inclusive). The time frame of 5 years was selected to provide us with sufficient data to examine the patterns in emerging topics and the number of tweets over time. Since the number of active users on the social media platform increased in recent years and we needed a large volume of data for topic modeling, we did not consider tweets posted before 2014. We selected the search keywords based on 3 studies on back pain [15,29,30]. These are detailed in Table 1. Search keywords were verified by our domain experts (FC, a rheumatologist; DU, a physiotherapist) who have extensive research and clinical expertise in the area of LBP. Selecting search keywords and an appropriate time frame are important considerations in the data collection process. The Monash University Human Research Ethics Committee approved this study (project ID 19738).

**Figure 1:**
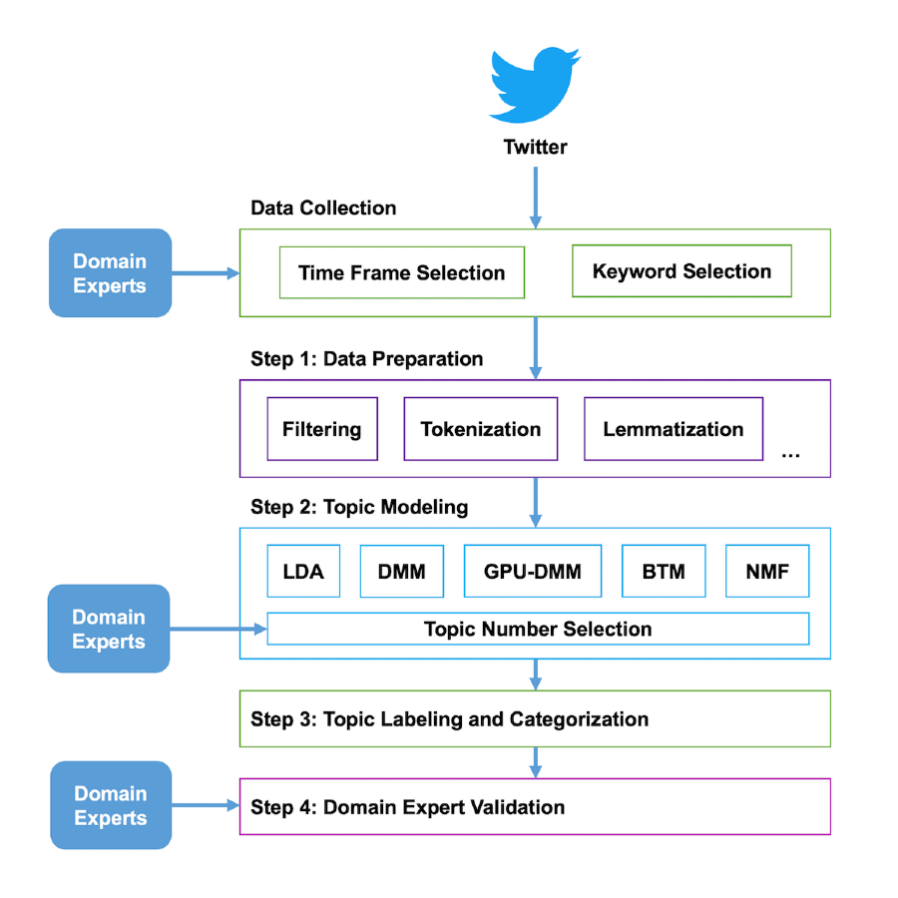
The overall data analysis workflow. The analysis consists of four steps: (1) data preprocessing, (2) thematic analysis using topic modelling, (3) topic labeling and categorization, and (4) domain expert validation. BTM: biterm topic model; DMM: Dirichlet multinomial mixture; GPU-DMM: General Pólya Urn Dirichlet Multinomial Mixture; LDA: latent Dirichlet allocation; NMF: nonnegative matrix factorization.

Our data processing and analysis consisted of 4 steps (see Figure 1).

**Table 1:** Keywords used to search tweets related to low back pain.

Source	Study purpose	Keywords	Total, n
Lee et al, 2016 [15]	To quantify the risks associated with a new tweet about back pain	“painful back,” “sore back,” “back started hurting,” “buggered my back,” “hurt my back,” “I’ve got backache,” “injured my back,” “my back hurts,” “I’ve got back pain,” “pain in my back,” “put my back out,” “my back is killing me”	12
Ahlwardt et al, 2014 [30]	To compare self-reported toothache experiences in tweets with those of backache, earache, and headache	“backache,” “back ache,” “back aches,” “back hurt,” “back hurting,” “back hurts,” “back killin’,” “back killing,” “back pain,” “back sore”	10
Campbell et al, 2013 [29]	A systematic review to study the influence of employment social support in nonspecific back pain	“lumbago,” “backache,” “back ache,” “back pain,” “low back ache,” “low back pain,” “lower back pains”	7

### Step 1: Data Preprocessing

We removed duplicates, retweets, URLs, and tweets related to marketing and advertisements, which reduced the data set from 7,892,210 to 2,825,645. We filtered the data further by removing tweets that did not contain first person pronouns [15]. As a result, the remaining data set size was 2,010,295.

We replaced contractions with their expanded forms (eg, “didn’t” to “did not”). We converted the HTML characters to ASCII characters and removed hashtags, Unicode strings (eg, “\u2026”), numbers, and punctuation. We replaced abbreviations, elongated words (eg, “gooood” to “good”), and emoticons and emojis with their equivalent English expressions. We then performed spelling correction, lowercasing, tokenization, and lemmatization, created n-grams, removed stop words (eg, common terms such as “the” and “is”). We again removed the duplicates and the remaining data set was 1,249,576 tweets.

After completing the abovementioned steps, we excluded tweets with less than three words because in topic modeling, the document size is important to achieve high accuracy [31]. This reduced the data set to 896,867 tweets.

### Step 2: Topic Modeling

Topic modeling is a technique used to provide a summary of a large collection of documents by extracting “topics” that represent the dominant themes [32]. It allows the uncovering of common, hidden themes from a corpus of text documents such as tweets. We tested 5 well-established topic modeling algorithms for detecting topics in a text-based corpus, namely latent Dirichlet allocation (LDA) [33], Dirichlet multinomial mixture (DMM) [34], GPU-DMM [35], biterm topic model (BTM) [36], and nonnegative matrix factorization (NMF) [37].

LDA is a generative probabilistic model that assumes each document can be represented by distribution over topics and each topic by distribution over words [33,38]. DMM is also a generative model but it assumes that each document is associated with one single topic [34,39]. GPU-DMM is an extended method of DMM that considers semantic similarity between words to provide semantic understanding of text documents and improve topic inference [35,40]. BTM uncovers topics by modeling the word co-occurrence patterns (ie, biterms) rather than using the document-level word co-occurrences [36,41]. NMF is able to learn the latent features in data using a nonnegative representation and improve latent semantic topic identification [37,42,43].

To use these models (except for NMF), we used a Java-based open-source library for short text topic modeling algorithms called STTM (version 1.8) [44], whereas for NMF we used the sklearn [45] library. For each approach, we performed a series of experiments ranging from 5 topics to 200 topics. We applied the 5 algorithms to the 896,867 tweets to determine the best model and the optimal number of topics.

Choosing the right number of topics is a crucial step in topic modeling because it can affect the accuracy of results. The quantitative approach computes the coherence score and perplexity, which helps in determining the optimal number of topics [46]. The coherence score measures the sum of the pairwise word-similarity scores of the words in the topic, using the pointwise mutual information (PMI) score [47]. Best collocation pairs usually have a high PMI. On the other hand, the qualitative approach requires humans and domain experts to examine the topics. Human judgment is extremely important because topic modeling uses a form of unsupervised learning.

As a quantitative approach, we calculated the coherence score of each model on different numbers of topics ranging from 5 to 200, based on the PMI score [47,48]. The coherence score was used to evaluate the quality of the topic-word distribution. LDA outperformed other approaches (ie, DMM, GPU-DMM, BTM, and NMF).

Additionally, we used a qualitative approach to select the most representative topics. We manually examined the topics, their top 20 terms, and a random sample of tweets in each topic. We also created a word cloud for each topic and evaluated word clouds and their sample tweets. We identified the number of topics that provided us with distinct and meaningful topics; if we exceeded this number of topics, we started to notice an increase in duplicates and overlapping topics. We used both quantitative and qualitative approaches to select the optimal number of topics.

### Step 3: Topic Labeling and Categorization

Topic labeling is a process of representing the meaning of a topic by assigning each topic a descriptive word or phrase [49]. Although automatic labeling approaches can reduce costs and time required, they are not able to achieve high semantic validity and accuracy [50,51]. In our study, we used the “eyeballing” method, which refers to reading and inspecting the top words in a topic and manually assigning a label [50]. We made sure that the results met the requirements of a “good” label: (1) semantically relevant, (2) meaningful, (3) representative, (4) adequate, and (5) understandable [34,49].

LDA assumes that each document (tweet) is a mix of topics with different proportions [33]. We were interested to examine tweets based on their dominant topic to gain a better understanding of the frequency of topics across all tweets. Therefore, we performed further analysis, and used the label of the dominant topic to represent each tweet, and then calculated the total number of tweets per topic.

To improve the results of thematic analysis, low-order topics can be grouped under broad, higher-order categories [52]. Higher-level categories can provide a better overview of the key topics discussed by individuals. To this end, after manual topic labeling, we performed topic categorization and assigned a category label to the topics that represented common themes. To identify the important and widely discussed categories, we then calculated the percentage of all tweets that corresponded to each individual category.

### Step 4: Domain Expert Validation

Two domain experts (FC, a rheumatologist; DU, a physiotherapist), actively working clinically and researchers in the area of LBP, independently examined the selected topics from the previous step where each topic included the top 20 words to determine face validity. As previously described, in topic modeling, the top words of each topic provide the description of that topic, thereby assisting the domain experts with inferring its meaning [49]. The experts then met to reconcile any differences and develop the final labels.

## Results

### Overview

The total number of collected tweets about LBP was 7,892,210 from 2,420,258 unique users from 2014 to 2018. The average number of words in each tweet increased from 2017 onward (Multimedia Appendix 1), in line with Twitter doubling the character limit of tweets from 140 to 280 characters as of November 2017 [53].

### Step 1: Data Preprocessing

After performing comprehensive data preprocessing, the final number of retained tweets was 896,867, which represents 11% (896,867/7,892,210) of the original raw data we collected, with a vocabulary size of 29,539. The minimum length of tweets was 4 words and the maximum length was 20 words.

### Step 2: Topic Modeling

After testing 5 topic modeling algorithms and the number of topics based on the coherence score and our manual examination, we selected the best model that included 60 topics, detected from 896,867 self-reported tweets about LBP. Multimedia Appendix 2 shows the coherence score of different models with a different number of topics ranging from 5 to 200. The best model was the LDA model with 60 topics, which had a coherence score of 0.562. Multimedia Appendix 3 shows the best model selected with 60 topics and their top 20 terms.

### Step 3: Topic Labeling and Categorization

The 60 topics were examined and manually given a topic label. The common and duplicate labels were then grouped into higher-order categories. Word clouds for the two categories of “pain regions” and “sleep” after combining the related topics are provided in Multimedia Appendix 4. The prevalence of the 60 manually labeled topics is presented in Multimedia Appendix 5.

### Step 4: Domain Expert Validation

**Figure 2:**
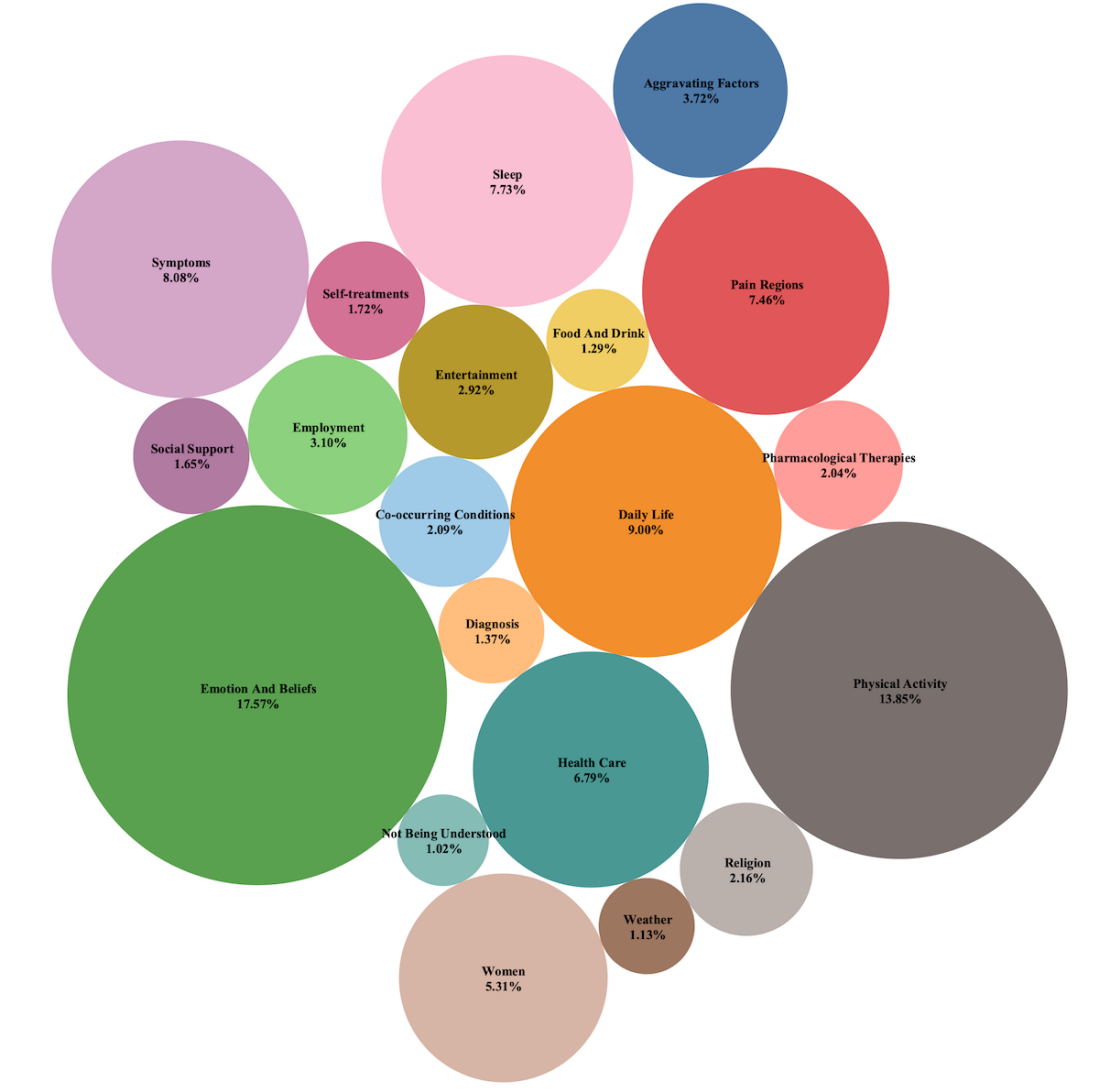
The 19 categories and their proportions based on all tweets posted from 2014 to 2018.

Independent examination of selected topics by two domain experts and reconciliation of any differences resulted in 19 contextual categories, with details presented in Multimedia Appendix 6. The total number of tweets within each of 19 contextual categories is presented in Figure 2, with more details in Multimedia Appendix 7. The “emotion and beliefs” category had the largest proportion of the total tweets, followed by “physical activity” and “daily life.” The lowest proportion of tweets belonged to the categories of “food and drink,” “weather,” and “not being understood.”

**Figure 3:**
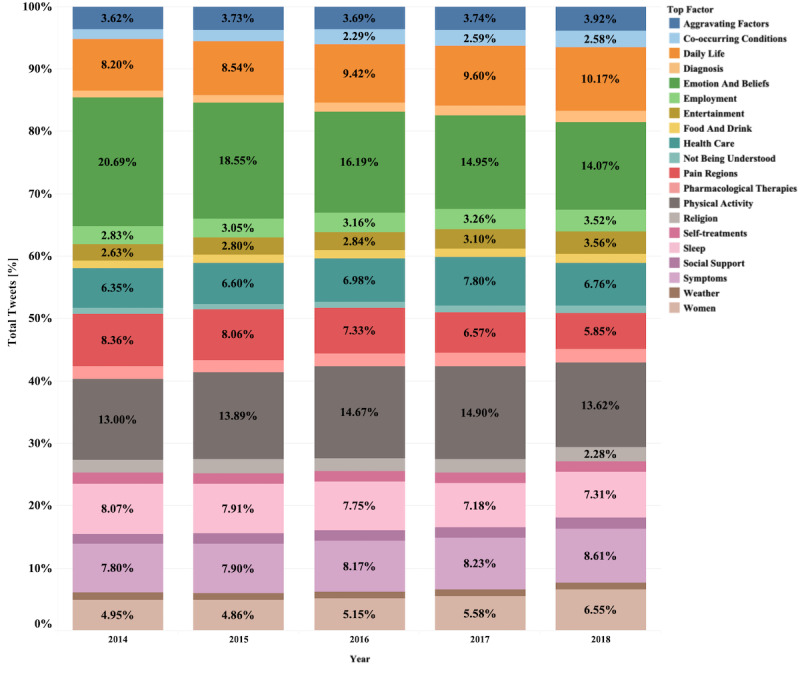
The proportions of 19 categories based on the dominant topic per year.

The proportion of tweets for each higher-level category over the years showed that all 19 categories had been discussed by individuals with relatively similar frequency every year (see Figure 3). However, the proportion of “emotion and beliefs” decreased from 2014 to 2018. The number of tweets about other categories, such as “aggravating factors” and “symptoms,” increased over that time period. An example of a tweet for each category is presented in Table 2 to illustrate the type of personal point of view related to each category.

**Table 2:** An example of tweets for each contextual category.

Categories	Examples of tweets
Emotion and beliefs	My back hurts, feeling sad because I wanna get up and do something ! I hate staying in bed :(
Physical activity	I did 6 miles on my exercise bike yesterday, felt really pleased with myself, and ate healthy. My back hurts today
Daily life symptoms	So my back hurts like hell and I can hardly sit here and do my hair. I hate it when my lower back hurts and sends shooting pains down my legs, making them ache and throb. Ugh.
Sleep	Every time I sleep in my sis guest bedroom my back hurts, that bed is not comfortable. I”d prolly be better off sleeping on the floor
Pain regions	today is not a good day. my back hurts, my shoulder hurts, my elbow is tingly, a little numb down to my hand and to top it off now my left knee hurts a little.
Health care	So I have found one good physio and one good chiropracter, both same price, who would you see if you had lower back pain?
Women	Being pregnant is literally taking everything out of me. I’m exhausted, my back is killing me and I stay moody…
Aggravating factors	Yesterday I tried doing a back flip on my trampoline. Now, every time I walk my back hurts. When I did the back flip I landed on my head.
Employment	Hurt my back at work yesterday and I’m working a full 12 hours tomorrow without getting paid. Lovin life right now.
Entertainment	Watching Cirque Du Soleil: Michael Jackson my back hurts just from watching it
Religion	Testimony Time! i want to give God the glory for healing me from a severe back pain
Co-occurring conditions	I don’t know if my back pain is causing depression or my depression is causing back pain…
Pharmacological therapies	I just took my very first Oxycodone for lower back pain. I think I’m in love. It didn’t just kill the pain. It assassinated it.
Self-treatments	Coconut oil epsom salt & vapor bath oil just soothed my back pain away
Social support	Told mom my back hurts she offered to rub my feet an back I have the best mom ever
Food and drink	my back is killing me cant get out ov bed but need coffee
Weather	I love cold weather but it’s really not helping with my back pain. Where is that warm summer weather attttttt.
Not being understood	OMG no one understands the pain I'm in right now. My back is killing me.

## Discussion

### Principal Results

In this study, we identified 60 specific topics from 896,867 tweets about LBP and grouped them into 19 categories that relate to contextual variables of LBP. The top category was “emotion and beliefs,” with 157,563/896,867 tweets (17.6%), followed by “physical activity” (124,251/896,867, 13.85%) and “daily life” (80,730/896,867, 9%), while “food and drink,” “weather,” and “not being understood” had the lowest proportions of tweets (11,551/896,867, 1.29%; 10,109/896,867, 1.13%; and 9180/896,867, 1.02%, respectively). There were 11 topics within the category of “emotion and beliefs”; of 157,563 tweets in this category, 113,562 (72%) expressed negative sentiment. Our results were consistent with the general findings from traditional study methods in the area of LBP but provided more in-depth detail on the context of LBP from the individual perspective.

### Comparison With Prior Work

Our study examined contextual variables to provide a novel insight into first-person perspectives of the LBP experience and confirmed the broad areas that have previously been identified using more traditional data collection methods from qualitative and quantitative studies. For example, psychosocial factors have an important role in LBP [54] and, from our analysis of tweets, “emotion and beliefs” was the most common topic we identified, with 157,563 of 896,867 tweets. This is consistent with LBP being widely recognized as a biopsychosocial condition, and growing evidence to show that psychological factors, such as beliefs and emotions, play an important role [55]. For instance, systematic reviews have highlighted that beliefs about back pain and negative consequences resulting from these beliefs are common across different countries and populations [56], and affect both treatment efficacy and prognosis [57]. Moreover, mass media campaigns that target negative beliefs have been implemented in an effort to influence how people manage their back pain on a population level [58]. Our study has also provided novel findings with respect to emotions. Although we found a range of emotions, from positive emotions (such as happy, love, or fun) to negative emotions (including hell, bad, or disgusting), the majority were found to be of negative affect. Although several studies have examined the role of specific emotions, such as anger [59,60], in LBP research, our understanding of the array of emotions experienced by individuals with back pain, specifically negative emotions, is limited.

Our study also highlighted areas related to the pain experience in individuals that have not been adequately explored in the literature but that play an important role in the effectiveness of LBP interventions and self-management behaviors, such as the “not being understood,” “religion,” and “food and drink” categories. We found that although the category of “not being understood” had the smallest proportion of tweets with a total of 9180 tweets, it had the top five words: “make,” “people,” ”stop,” “thing,” and “complain.” This is consistent with a previous systematic scoping review that examined what patients want from their medical care, which reported that patients felt misunderstood and wanted legitimation of their LBP [11]. Patients with LBP report negative social stereotyping from health care professionals, family and friends, and the community [61] and that they are dissatisfied with the inadequate advice they receive from medical practitioners and have identified an unmet need for care providers that show more understanding and empathy [11].

The category of “food and drink” is novel and interesting. The tweets included words relating to the type of food (eg, pizza, chocolate, cookies and cream), mealtimes (such as breakfast and lunch), and the process of bringing or making food. Although they reflect important daily habits of eating and drinking, they may also highlight issues around pain affecting an individual’s capacity to eat and drink and/or problems associated with weight and in particular obesity [62], which is a major public health issue [63].

There are well-described sex differences in the prevalence of back pain [64]. Analysis of tweets identified 3 topics under the “women” category including “motherhood,” “large breasts problem,” and “female health complaints.” LBP has been reported in more than two-thirds of pregnant women [65]. Improving psychological well-being, physical fitness, and general well-being may reduce LBP in women [65-67]. The topics identified in tweets may provide more direction in relation to the personal topics that warrant further examination (eg, the potential effect of “large breasts problem” and whether this is a cause of LBP or a potential confounding variable). Identifying possible mechanisms for the association with topics such as “motherhood” or “female health complaints” could also help with understanding whether these associations are due to psychosocial factors or biomechanical factors such as the lifting and carrying of children. Understanding the context of LBP could offer valuable insights into how people with LBP view and experience their condition; this could lead to the identification of new areas of research in exploring the causes of LBP, as well as the opportunity to identify areas of potential misinformation that need to be addressed.

### Limitations

There are some limitations to our study. Although the keywords were taken from existing studies about LBP and approved by domain experts, some keywords, such as “back hurt” and “back pain,” were very broad. Therefore, the data collected might not have been specific to LBP. Selection of the right keywords in Twitter data analysis is very important to avoid unrelated data that could reduce the accuracy of results. Filtering and cleaning of Twitter data is also crucial for achieving high accuracy of results. In our study, we performed vigorous data cleaning, but our manual examination showed that there was a group of tweets that contained a few lines from the lyrics of a famous hip-hop song (Bad and Boujee) by Migos. These lines included “…So my money makin' my back ache.” One of our search keywords was “back ache.” Although there are many tools and methods available to automatically perform data cleaning, it is always necessary to manually inspect the results.

Twitter users tend to be younger and might not represent the general population; therefore, the results must be carefully interpreted [68]. Similar to other social media studies in health care, we cannot verify that individuals who tweeted about LBP were actually real patients [15]. However, the filtering based on first-person pronouns (eg, I, my, or mine) that we performed is likely to have reduced this.

To determine the optimal number of topics, we used the coherence score, a widely used method, and then manually examined and compared the models. This process can be further improved by using other measures such as heuristic approaches [69] or perplexity measures [70].

We also recognize that manual labeling of topics can be subjective. Two domain experts with extensive knowledge were involved in the labeling and examination of selected topics but future work in this area could involve a greater number of and more diverse domain experts to further reduce this subjectivity.

### Conclusions

Our findings provided useful insights into individuals’ beliefs and perspectives regarding their needs and concerns related to LBP that complement the information available in the literature. Considering the contextual factors identified in this study rather than simply focusing on a biomedical model of LBP could address the needs of patients more holistically, help with improving LBP outcomes, and increase patient satisfaction. These findings have the potential to assist health care providers and clinicians with developing more effective, personalized therapies for LBP. There is also the potential to use social media to identify any major changes in community beliefs and needs regarding LBP that can be addressed in a timelier manner.

## Appendix

Multimedia Appendix 1 The average number of words in tweets per year.

Multimedia Appendix 2 Coherence score for latent Dirichlet allocation, Dirichlet multinomial mixture (DMM), General Pólya Urn Dirichlet Multinomial Mixture (GPU-DMM), biterm topic model, and nonnegative matrix factorization with number of topics 5-200 .

Multimedia Appendix 3 The best model selected with 60 topics and their top 20 terms.

Multimedia Appendix 4 Word clouds for the pain region and sleep categories.

Multimedia Appendix 5 Total number of tweets per each topic manually labelled.

Multimedia Appendix 6 The 19 contextual categories related to low back pain.

Multimedia Appendix 7 The total and percentage of tweets for each contextual category.

## Abbreviations

API: application programming interface
BTM: biterm topic model
DMM: Dirichlet multinomial mixture
GPU-DMM: General Pólya Urn Dirichlet Multinomial Mixture
LBP: low back pain
LDA: latent Dirichlet allocation
NMF: nonnegative matrix factorization
PMI: pointwise mutual information
STTM: short text topic modeling algorithm

## References

[1] Hoy D, March L, Brooks P, Blyth F, Woolf A, Bain C, Williams G, Smith E, Vos T, Barendregt J, Murray C, Burstein R, Buchbinder R (2014). The global burden of low back pain: estimates from the Global Burden of Disease 2010 study. Ann Rheum Dis.

[2] Global Burden of Disease Study 2013 Collaborators (2015). Global, regional, and national incidence, prevalence, and years lived with disability for 301 acute and chronic diseases and injuries in 188 countries, 1990-2013: a systematic analysis for the Global Burden of Disease Study 2013. Lancet.

[3] Rubin DI (2007). Epidemiology and risk factors for spine pain. Neurol Clin.

[4] Hoy D, Brooks P, Blyth F, Buchbinder R (2010). The Epidemiology of low back pain. Best Pract Res Clin Rheumatol.

[5] Guo HR, Tanaka S, Halperin WE, Cameron LL (1999). Back pain prevalence in US industry and estimates of lost workdays. Am J Public Health.

[6] Katz JN (2006). Lumbar disc disorders and low-back pain: socioeconomic factors and consequences. J Bone Joint Surg Am.

[7] Mafi JN, McCarthy EP, Davis RB, Landon BE (2013). Worsening trends in the management and treatment of back pain. JAMA Intern Med.

[8] Chou L, Cicuttini FM, Urquhart DM, Anthony SN, Sullivan K, Seneviwickrama M, Briggs AM, Wluka AE (2018). People with low back pain perceive needs for non-biomedical services in workplace, financial, social and household domains: a systematic review. J Physiother.

[9] Lim YZ, Chou L, Au RT, Seneviwickrama KMD, Cicuttini FM, Briggs AM, Sullivan K, Urquhart DM, Wluka AE (2019). People with low back pain want clear, consistent and personalised information on prognosis, treatment options and self-management strategies: a systematic review. J Physiother.

[10] De Souza LH, Frank AO (2000). Subjective pain experience of people with chronic back pain. Physiother Res Int.

[11] Chou L, Ranger TA, Peiris W, Cicuttini FM, Urquhart DM, Sullivan K, Seneviwickrama K, Briggs AM, Wluka AE (2018). Patients' perceived needs of health care providers for low back pain management: a systematic scoping review. Spine J.

[12] Symonds TL, Burton AK, Tillotson KM, Main CJ (1995). Absence resulting from low back trouble can be reduced by psychosocial intervention at the work place. Spine (Phila Pa 1976).

[13] Waddell G, Newton M, Henderson I, Somerville D, Main C (1993). A Fear-Avoidance Beliefs Questionnaire (FABQ) and the role of fear-avoidance beliefs in chronic low back pain and disability. Pain.

[14] Delir Haghighi P, Kang Y, Buchbinder R, Burstein F, Whittle S (2017). Investigating Subjective Experience and the Influence of Weather Among Individuals With Fibromyalgia: A Content Analysis of Twitter. JMIR Public Health Surveill.

[15] Lee H, McAuley JH, Hübscher M, Allen HG, Kamper SJ, Moseley GL (2016). Tweeting back: predicting new cases of back pain with mass social media data. J Am Med Inform Assoc.

[16] Asghar MZ, Ahmad S, Qasim M, Zahra SR, Kundi FM (2016). SentiHealth: creating health-related sentiment lexicon using hybrid approach. Springerplus.

[17] Raghupathi W, Raghupathi V (2014). Big data analytics in healthcare: promise and potential. Health Inf Sci Syst.

[18] Bian J, Topaloglu U, Yu F (2012). Towards Large-scale Twitter Mining for Drug-related Adverse Events. SHB12 (2012).

[19] Pershad Y, Hangge P, Albadawi H, Oklu R (2018). Social Medicine: Twitter in Healthcare. J Clin Med.

[20] Aichner T, Jacob F (2015). Measuring the Degree of Corporate Social Media Use. International Journal of Market Research.

[21] Sinnenberg L, Buttenheim AM, Padrez K, Mancheno C, Ungar L, Merchant RM (2017). Twitter as a Tool for Health Research: A Systematic Review. Am J Public Health.

[22] Jayaraman PP, Forkan ARM, Morshed A, Haghighi PD, Kang Y (2019). Healthcare 4.0: A review of frontiers in digital health. WIREs Data Mining Knowl Discov.

[23] Goh T, Delir Haghighi P, Burstein F, Buchbinder R (2015). Developing a Contextual Model towards Understanding Low Back Pain. Proceedings of the 19th Pacific Asia Conference on Information Systems.

[24] Hsieh H, Shannon SE (2005). Three approaches to qualitative content analysis. Qual Health Res.

[25] Hewis J (2015). Do MRI Patients Tweet? Thematic Analysis of Patient Tweets About Their MRI Experience. J Med Imaging Radiat Sci.

[26] Prier K, Smith M, Giraud-Carrier C, Hanson C, Salerno J, Yang SJ, Nau D, Chai SK (2011). Identifying Health-Related Topics on Twitter: An Exploration of Tobacco-Related Tweets as a Test Topic. Social Computing, Behavioral-Cultural Modeling and Prediction.

[27] TWINT - Twitter Intelligence Tool. GitHub.

[28] Xavier C, Souza M, Roesler V, Barrére E, Willrich R (2020). A Basic Approach for Extracting and Analyzing Data from Twitter. Special Topics in Multimedia, IoT and Web Technologies.

[29] Campbell P, Wynne-Jones G, Muller S, Dunn KM (2013). The influence of employment social support for risk and prognosis in nonspecific back pain: a systematic review and critical synthesis. Int Arch Occup Environ Health.

[30] Ahlwardt K, Heaivilin N, Gibbs J, Page J, Gerbert B, Tsoh JY (2014). Tweeting about pain: comparing self-reported toothache experiences with those of backaches, earaches and headaches. J Am Dent Assoc.

[31] Jian T, Zhaoshi M, Xuanlong N, Qiaozhu M, Ming Z (2014). Understanding the limiting factors of topic modeling via posterior contraction analysis.

[32] Blei D, Carin L, Dunson D (2010). Probabilistic Topic Models: A focus on graphical model design and applications to document and image analysis. IEEE Signal Process Mag.

[33] Blei D, Ng A, Jordan M (2003). Latent dirichllocation. Journal of Machine Learning Research.

[34] Nigam K, Mccallum AK, Thrun S, Mitchell T (2000). Text Classification from Labeled and Unlabeled Documents using EM. Machine Learning.

[35] Li C, Wang H, Zhang Z, Sun A, Ma Z (2016). Topic Modeling for Short Texts with Auxiliary Word Embeddings. SIGIR '16: Proceedings of the 39th International ACM SIGIR conference on Research and Development in Information Retrieval.

[36] Cheng X, Yan X, Lan Y, Guo J (2014). BTM: Topic Modeling over Short Texts. IEEE Trans Knowl Data Eng.

[37] Lee D, Seung S (2000). Algorithms for Non-negative Matrix Factorization.

[38] Chandrasekaran R, Mehta V, Valkunde T, Moustakas E (2020). Topics, Trends, and Sentiments of Tweets About the COVID-19 Pandemic: Temporal Infoveillance Study. J Med Internet Res.

[39] Surian D, Nguyen DQ, Kennedy G, Johnson M, Coiera E, Dunn AG (2016). Characterizing Twitter Discussions About HPV Vaccines Using Topic Modeling and Community Detection. J Med Internet Res.

[40] Liang W, Feng R, Liu X, Li Y, Zhang X (2018). GLTM: A Global and Local Word Embedding-Based Topic Model for Short Texts. IEEE Access.

[41] Mackey T, Kalyanam J, Klugman J, Kuzmenko E, Gupta R (2018). Solution to Detect, Classify, and Report Illicit Online Marketing and Sales of Controlled Substances via Twitter: Using Machine Learning and Web Forensics to Combat Digital Opioid Access. J Med Internet Res.

[42] Odlum M, Yoon S, Broadwell P, Brewer R, Kuang D (2018). How Twitter Can Support the HIV/AIDS Response to Achieve the 2030 Eradication Goal: In-Depth Thematic Analysis of World AIDS Day Tweets. JMIR Public Health Surveill.

[43] Wang Y, Zhang Y (2013). Nonnegative Matrix Factorization: A Comprehensive Review. IEEE Trans Knowl Data Eng.

[44] STTM: A Library of Short Text Topic Modeling. GitHub.

[45] scikit-learn: Machine Learning in Python.

[46] Chang J, Gerrish S, Wang C, Boyd-graber J, Blei D (2009). Reading tea leaves: how humans interpret topic models. https://papers.nips.cc/paper/2009/hash/f92586a25bb3145facd64ab20fd554ff-Abstract.html.

[47] Church K, Hanks P (1990). Word association norms, mutual information, and lexicography. Computational Linguistics.

[48] Newman D, Bonilla E, Buntine W (2011). Improving topic coherence with regularized topic models. https://papers.nips.cc/paper/2011/hash/5ef698cd9fe650923ea331c15af3b160-Abstract.html.

[49] Allahyari M, Pouriyeh S, Kochut K, Reza H (2017). A Knowledge-based Topic Modeling Approach for Automatic Topic Labeling. ijacsa.

[50] Morstatter F, Liu H (2017). In Search of Coherence and Consensus: Measuring the Interpretability of Statistical Topics. Journal of Machine Learning Research.

[51] Grimmer J, Stewart BM (2017). Text as Data: The Promise and Pitfalls of Automatic Content Analysis Methods for Political Texts. Political Analysis.

[52] Nowell LS, Norris JM, White DE, Moules NJ (2017). Thematic Analysis: Striving to Meet the Trustworthiness Criteria. International Journal of Qualitative Methods.

[53] Boot AB, Tjong Kim Sang E, Dijkstra K, Zwaan RA (2019). How character limit affects language usage in tweets. Palgrave Commun.

[54] Pincus T, Burton AK, Vogel S, Field AP (2002). A systematic review of psychological factors as predictors of chronicity/disability in prospective cohorts of low back pain. Spine (Phila Pa 1976).

[55] Maher C, Underwood M, Buchbinder R (2017). Non-specific low back pain. Lancet.

[56] Morton L, de Bruin M, Krajewska M, Whibley D, Macfarlane G (2019). Beliefs about back pain and pain management behaviours, and their associations in the general population: A systematic review. Eur J Pain.

[57] Wertli MM, Rasmussen-Barr E, Held U, Weiser S, Bachmann LM, Brunner F (2014). Fear-avoidance beliefs-a moderator of treatment efficacy in patients with low back pain: a systematic review. Spine J.

[58] Urquhart DM, Bell RJ, Cicuttini FM, Cui J, Forbes A, Davis SR (2008). Negative beliefs about low back pain are associated with high pain intensity and high level disability in community-based women. BMC Musculoskelet Disord.

[59] Bruehl S, Liu X, Burns J, Chont M, Jamison R (2012). Associations between daily chronic pain intensity, daily anger expression, and trait anger expressiveness: an ecological momentary assessment study. Pain.

[60] Burns JW, Quartana P, Bruehl S (2011). Anger suppression and subsequent pain behaviors among chronic low back pain patients: moderating effects of anger regulation style. Ann Behav Med.

[61] Slade SC, Molloy E, Keating JL (2009). Stigma experienced by people with nonspecific chronic low back pain: a qualitative study. Pain Med.

[62] Chou L, Brady S, Urquhart D, Teichtahl AJ, Cicuttini FM, Pasco JA, Brennan-Olsen SL, Wluka AE (2016). The Association Between Obesity and Low Back Pain and Disability Is Affected by Mood Disorders: A Population-Based, Cross-Sectional Study of Men. Medicine (Baltimore).

[63] Agha M, Agha R (2017). The rising prevalence of obesity: part A: impact on public health. Int J Surg Oncol (N Y).

[64] Wu A, March L, Zheng X, Huang J, Wang X, Zhao J, Blyth FM, Smith E, Buchbinder R, Hoy D (2020). Global low back pain prevalence and years lived with disability from 1990 to 2017: estimates from the Global Burden of Disease Study 2017. Ann Transl Med.

[65] Liddle S, Pennick V (2015). Interventions for preventing and treating low-back and pelvic pain during pregnancy. Cochrane Database Syst Rev.

[66] Brady SR, Hussain SM, Brown WJ, Heritier S, Wang Y, Teede H, Urquhart DM, Cicuttini FM (2018). Course and Contributors to Back Pain in Middle-aged Women Over 9 Years: Data From the Australian Longitudinal Study on Women's Health. Spine (Phila Pa 1976).

[67] Ng SK, Cicuttini FM, Davis SR, Bell R, Botlero R, Fitzgibbon BM, Urquhart DM (2018). Poor general health and lower levels of vitality are associated with persistent, high-intensity low back pain and disability in community-based women: A prospective cohort study. Maturitas.

[68] Zhang H, Wheldon C, Dunn A, Tao C, Huo Ji, Zhang R, Prosperi M, Guo Y, Bian J (2020). Mining Twitter to assess the determinants of health behavior toward GPU-DMM: General Pólya Urn Dirichlet Multinomial Mixturehuman papillomavirus vaccination in the United States. J Am Med Inform Assoc.

[69] Zhao W, Chen JJ, Perkins R, Liu Z, Ge W, Ding Y, Zou W (2015). A heuristic approach to determine an appropriate number of topics in topic modeling. BMC Bioinformatics.

[70] Wallach H, Murray I, Salakhutdinov R, Mimno D (2009). Evaluation methods for topic models. Proceedings of the 26th Annual International Conference on Machine Learning.

