# Supplementary figures and images for: Investigating Individuals’ Perceptions Regarding the Context Around the Low Back Pain Experience: Topic Modeling Analysis of Twitter Data
Source: J Med Internet Res. 2021 Dec 23;23(12):e26093. doi: 10.2196/26093 (PMC8738994; doi:10.2196/26093)

**Multimedia Appendix 1 - The average number of words in tweets per year**


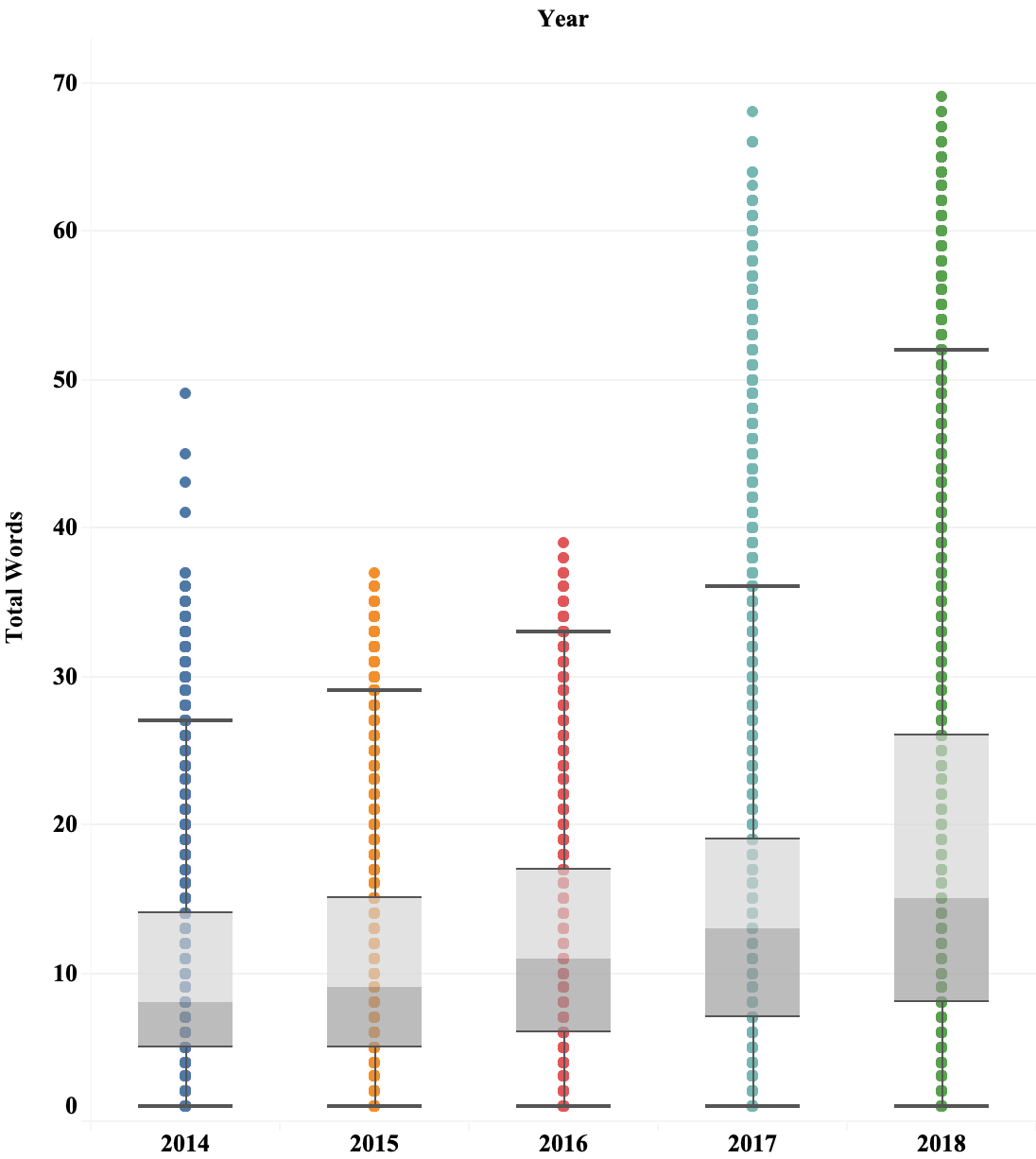

Supplement: Multimedia Appendix 1 [file jmir_v23i12e26093_app1.docx]

**Multimedia Appendix 2: Coherence Score for LDA, DMM, GPU-DMM, BTM, and NMF with number of topics 5- 200**


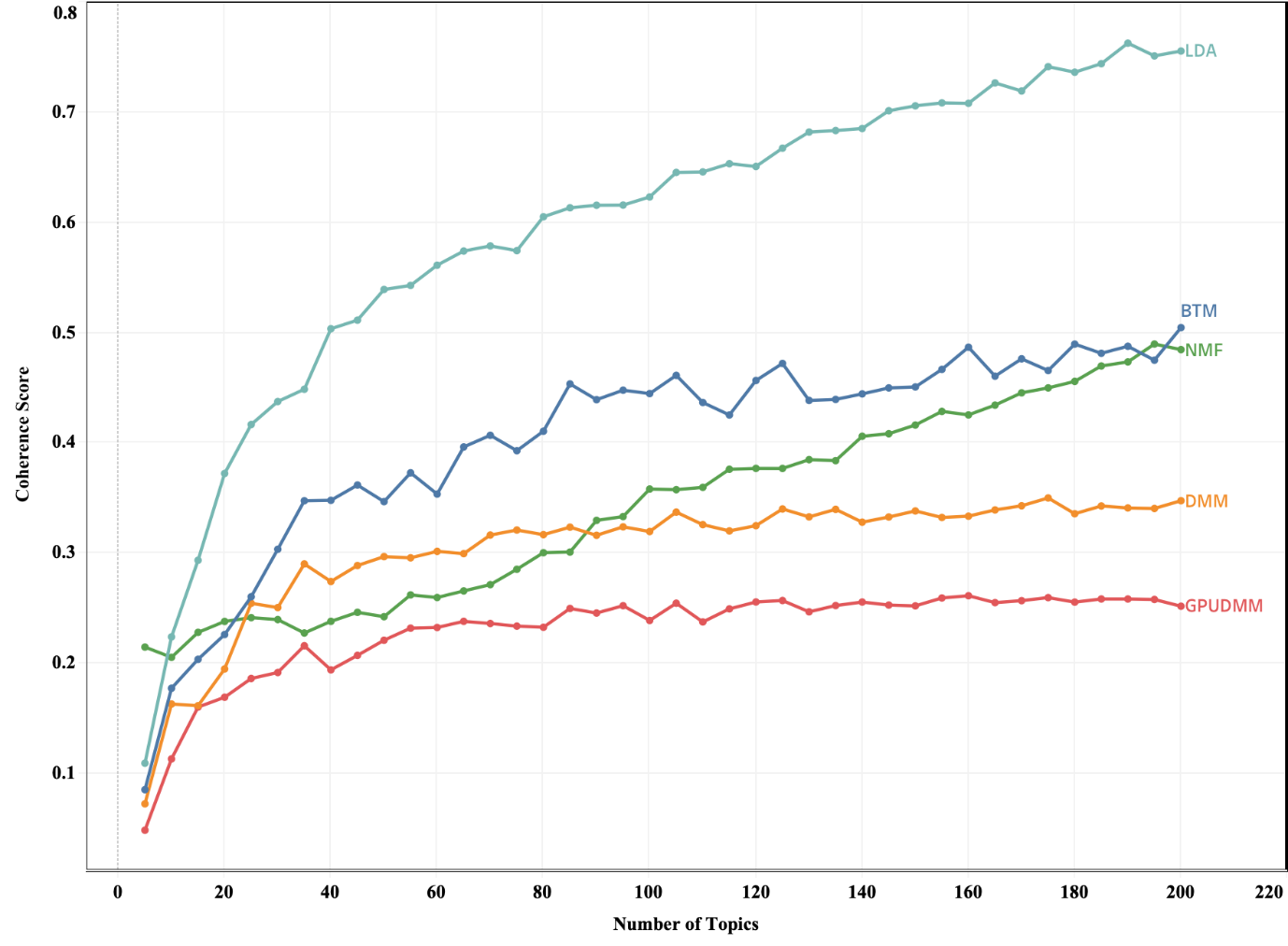

Supplement: Multimedia Appendix 2 [file jmir_v23i12e26093_app2.docx]

**Multimedia Appendix 4 - Word clouds for the pain region and sleep categories.**


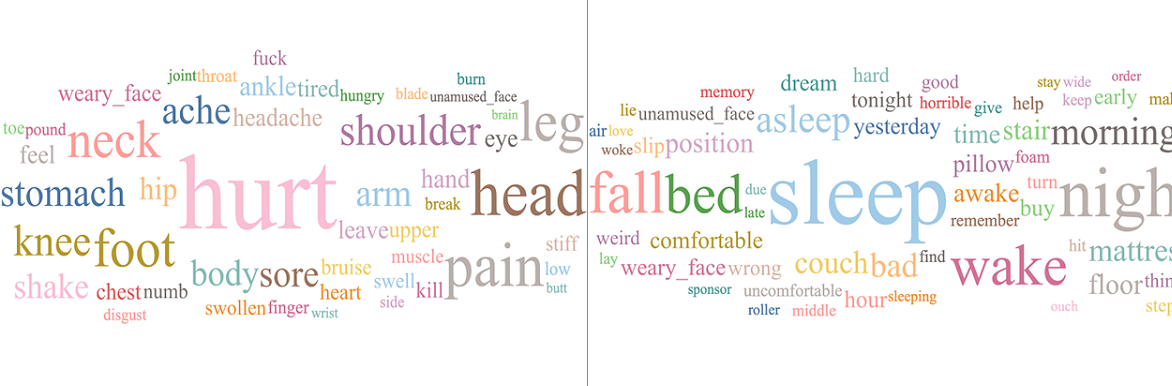

Supplement: Multimedia Appendix 4 [file jmir_v23i12e26093_app4.docx]
