# Supplementary material for: Investigating Individuals’ Perceptions Regarding the Context Around the Low Back Pain Experience: Topic Modeling Analysis of Twitter Data
Source: J Med Internet Res. 2021 Dec 23;23(12):e26093. doi: 10.2196/26093 (PMC8738994; doi:10.2196/26093)
Supplement: Multimedia Appendix 3 [file jmir_v23i12e26093_app3.docx]

**Multimedia Appendix 3: The best model selected with 60 topics and their top 20 terms**

| **Topic 0** | **Topic 1** | **Topic 2** | **Topic 3** | **Topic 4** |
| --- | --- | --- | --- | --- |
| good | god | week | car | muscle |
| today | today | day | drive | posture |
| feel | pray | year | ride | exercise |
| morning | lord | ago | accident | pull |
| bad | love | time | seat | low |
| wake | friend | month | hour | stretch |
| thing | hope | past | hit | hip |
| news | heal | couple | bus | core |
| idea | dear | start | home | reduce |
| great | good | finally | long | improve |
| pretty | bless | work | train | body |
| start | jesus | severe | end | prevent |
| yesterday | prayer | end | trip | spine |
| hope | folded_hand | due | road | poor |
| bit | give | chiropractor | bike | tight |
| work | send | surgery | today | help |
| mood | severe | think | neck | injury |
| feeling | heart | pain | horse | tip |
| night | fix | pretty | yesterday | cause |
| lot | thankful | remember | truck | strong |
| **Topic 5** | **Topic 6** | **Topic 7** | **Topic 8** | **Topic 9** |
| put | sleep | pain | fall | laugh |
| wear | night | chronic | asleep | loud |
| make | bed | stress | night | ass |
| today | position | suffer | bad | hard |
| shoe | couch | problem | stair | fuck |
| bar | floor | anxiety | wake | shit |
| work | comfortable | disc | yesterday | kick |
| time | wake | arthritis | slip | damn |
| thing | bad | issue | hard | bad |
| high | tonight | severe | couch | man |
| heel | wrong | cause | morning | make |
| bend | weird | depression | step | bitch |
| low | uncomfortable | spinal | floor | dumb |
| brace | weary_face | joint | ice | stop |
| tight | lie | oil | weary_face | fat |
| leave | find | year | remember | bust |
| top | pillow | migraine | bed | yeah |
| pant | lay | headache | hit | beat |
| stop | unamused_face | sciatica | ouch | funny |
| find | sleeping | neck | unamused_face | hell |
| **Topic 10** | **Topic 11** | **Topic 12** | **Topic 13** | **Topic 14** |
| pain | bed | weary_face | dance | baby |
| pill | lay | bad | show | love |
| take | lie | tired_face | tonight | pregnant |
| give | bad | pensive_face | love | pregnancy |
| killer | pad | unamused_face | fun | week |
| painkiller | heating | confounded_face | hut | girl |
| doctor | today | crying_face | dancing | boy |
| medicine | floor | pistol | party | kick |
| drug | move | kill | worth | give |
| pop | literally | pouting_face | hard | miss |
| ibuprofen | watch | sleepy_face | practice | time |
| muscle | couch | thumbs_down | huh | month |
| work | cry | flushed_face | yamaha | make |
| hope | flat | confused_face | miss | son |
| help | stuck | angry_face | concert | child |
| kick | rest | fuck | music | ready |
| strong | stay | hate | band | rub |
| high | make | org | weekend | care |
| shot | weary_face | oncoming_fist | song | eye |
| bed | lazy | disgusted | cheer | wait |
| **Topic 15** | **Topic 16** | **Topic 17** | **Topic 18** | **Topic 19** |
| big | hurt | hair | yoga | hot |
| boob | head | today | stretch | bath |
| girl | foot | hour | exercise | water |
| make | leg | finish | relieve | shower |
| small | neck | break | great | heat |
| ass | knee | draw | relief | drink |
| problem | stomach | cut | tip | bottle |
| breast | arm | hand | help | cold |
| hate | shoulder | make | check | nice |
| huge | hip | time | pose | ice |
| bar | throat | art | free | pack |
| give | eye | spend | suffer | put |
| people | heart | take | class | icy |
| reduction | chest | paint | minute | help |
| time | body | finger | ease | relax |
| prob | ankle | finally | find | warm |
| size | hand | work | simple | wine |
| titty | wrist | nail | health | tub |
| fit | butt | wash | video | take |
| suck | brain | eye | rid | time |
| **Topic 20** | **Topic 21** | **Topic 22** | **Topic 23** | **Topic 24** |
| mon | leg | tomorrow | cramp | home |
| tell | foot | today | period | work |
| dad | sore | work | hate | bed |
| call | hurt | hope | headache | today |
| ask | arm | tonight | bad | ready |
| complain | knee | week | stomach | stay |
| sum | hand | stream | pain | wait |
| talk | ankle | wait | mother | weary_face |
| brother | bruise | chiropractor | feel | tired |
| smoke | body | appointment | fuck | finally |
| stop | swell | friday | mood | bad |
| sister | numb | morning | month | early |
| doctor | swollen | monday | low | tomorrow |
| friend | feel | guy | kill | leave |
| give | shoulder | start | nausea | school |
| phone | hip | doctor | start | lie |
| yeah | toe | weekend | pregnant | glad |
| weed | leave | call | girl | disgust |
| parent | finger | make | die | tonight |
| bitch | break | rest | horrible | rest |
| **Topic 25** | **Topic 26** | **Topic 27** | **Topic 28** | **Topic 29** |
| heavy | bad | sit | night | work |
| carry | weary_face | stand | good | hour |
| lift | sleep | long | happy | today |
| today | side | hour | today | shift |
| bag | pensive_face | straight | love | job |
| dead | tired_face | time | sleep | foot |
| box | unamused_face | bad | time | leave |
| move | low | lie | bed | tired |
| stuff | wrong | walk | christmas | tomorrow |
| book | leave | minute | tired | day |
| pick | damn | lay | goodnight | home |
| lifting | crying_face | position | tonight | half |
| work | stomach | min | birthday | kill |
| yesterday | evil_monkey | make | friend | hard |
| ass | lower | matter | fun | money |
| thing | wake | anymore | kiss | weary_face |
| weight | confounded_face | today | rest | call |
| school | confused_face | floor | bye | tonight |
| put | disgust | literally | hope | early |
| pack | man | day | present | spend |
| **Topic 30** | **Topic 31** | **Topic 32** | **Topic 33** | **Topic 34** |
| life | dog | doctor | massage | kidney |
| people | jump | call | give | grin |
| live | face | pay | weary_face | nerve |
| pain | phone | care | bad | doctor |
| experience | hit | chronic | rub | pain |
| chronic | open | medical | good | kiss |
| thing | throw | year | body | think |
| deal | door | people | full | low |
| suffer | cat | patient | pay | stone |
| point | flip | tell | need | severe |
| time | eye | hospital | deep | attack |
| year | drop | chiropractor | boyfriend | blood |
| amount | walk | severe | pensive_face | die |
| constant | run | surgery | nice | heart |
| problem | kid | insurance | tired_face | turn |
| fact | turn | visit | smirking_face | hope |
| daily | wall | card | sea | cancer |
| age | time | health | eye | hospital |
| understand | hard | office | real | infection |
| everyday | push | check | raising_hand | week |
| **Topic 35** | **Topic 36** | **Topic 37** | **Topic 38** | **Topic 39** |
| feel | run | bed | school | therapy |
| sick | arm | mattress | today | physical |
| make | today | sleep | tomorrow | chiropractic |
| bad | workout | buy | class | treatment |
| feeling | weight | time | work | chronic |
| today | week | pillow | finish | care |
| stomach | yesterday | thing | study | study |
| throw | lose | wake | tired | suffer |
| shit | squat | help | homework | treat |
| headache | leg | foam | write | patient |
| nauseous | time | make | hour | relief |
| disgust | deadline | air | start | chiropractor |
| hate | walk | memory | exam | acupuncture |
| good | mile | give | week | pain |
| horrible | training | roll | miss | find |
| crap | start | love | test | show |
| dizzy | gain | roller | final | health |
| die | lift | morning | stress | neck |
| awful | stop | sponsor | paper | great |
| weak | low | order | break | recommend |
| **Topic 40** | **Topic 41** | **Topic 42** | **Topic 43** | **Topic 44** |
| eat | sleep | pain | headache | bad |
| make | night | neck | sore | cry |
| food | wake | shoulder | throat | weary_face |
| today | morning | leg | feel | literally |
| coffee | awake | knee | cough | crying_face |
| drink | hour | headache | nose | tired_face |
| bring | bed | ache | sick | god |
| dinner | dream | hip | cold | tear |
| ice | early | body | eye | confounded_face |
| tea | turn | upper | fever | damn |
| cook | good | stiff | ache | bandage |
| cream | time | leave | body | org |
| pizza | horrible | muscle | hurt | pensive_face |
| chocolate | middle | chest | tooth | sleepy_face |
| lunch | keep | arm | ear | real |
| chicken | wide | feel | head | fuck |
| cookie | late | low | stomach | lower |
| hungry | stay | side | fly | make |
| cake | due | joint | stop | freak |
| breakfast | woke | blade | chest | die |
| **Topic 45** | **Topic 46** | **Topic 47** | **Topic 48** | **Topic 49** |
| head | year | play | today | sit |
| hurt | feel | game | cold | chair |
| shake | man | carry | snow | hour |
| ache | lady | team | weather | desk |
| stomach | age | ball | make | today |
| foot | woman | watch | yesterday | work |
| tired | body | basketball | shovel | computer |
| weary_face | young | win | rain | office |
| kill | old | tonight | sun | long |
| leg | time | football | time | stand |
| fuck | turn | today | garden | laptop |
| eye | knee | golf | weekend | table |
| body | person | playing | hate | front |
| neck | swear | yesterday | nice | uncomfortable |
| pound | grandma | time | summer | posture |
| heart | bad | practice | winter | lean |
| burn | problem | tiger | walk | straight |
| hungry | officially | soccer | great | spend |
| disgust | complain | man | work | make |
| unamused_face | middle | hit | love | hunch |
| **Topic 50** | **Topic 51** | **Topic 52** | **Topic 53** | **Topic 54** |
| bad | feel | make | clean | tired |
| move | bad | people | room | sleep |
| walk | time | stop | house | hungry |
| barely | spine | thing | move | feel |
| literally | pain | complain | today | sleepy |
| cry | breathe | bitch | floor | headache |
| weary_face | breath | talk | stuff | fuck |
| breathe | hard | think | hour | hell |
| hurt | move | lot | put | unamused_face |
| pain | die | shit | wash | super |
| point | sharp | hear | finish | disgust |
| fuck | stab | hate | spend | today |
| lower | sneeze | tweet | laundry | cold |
| badly | crack | man | cleaning | sick |
| make | low | yeah | living | exhausted |
| bend | rib | kind | kitchen | weary_face |
| tired_face | literally | real | clothe | mood |
| properly | make | sense | bathroom | stress |
| feel | rip | person | dish | sad |
| idea | body | sound | finally | pensive_face |
| **Topic 55** | **Topic 56** | **Topic 57** | **Topic 58** | **Topic 59** |
| fuck | today | watch | shit | today |
| life | upside | write | fuck | work |
| bad | down_face | video | damn | long |
| hate | bad | read | weary_face | learn |
| fucking | wake | movie | bad | yesterday |
| bitch | love | story | man | rest |
| shit | life | love | holy | week |
| suck | great | show | unamused_face | lot |
| man | start | word | hell | great |
| damn | feel | listen | ass | fun |
| god | work | post | god | weekend |
| hell | time | book | turf | tomorrow |
| disgust | morning | guy | feel | start |
| unamused_face | fun | star | bitch | day |
| stupid | cool | life | brush | busy |
| weary_face | fine | make | fucking | time |
| dude | die | music | tired_face | hard |
| scoliosis | pretty | time | outta | good |
| disgusted | literally | episode | crap | guess |
| pouting_face | reason | tweet | swear | hope |
