# Supplementary material for: Investigating Individuals’ Perceptions Regarding the Context Around the Low Back Pain Experience: Topic Modeling Analysis of Twitter Data
Source: J Med Internet Res. 2021 Dec 23;23(12):e26093. doi: 10.2196/26093 (PMC8738994; doi:10.2196/26093)
Supplement: Multimedia Appendix 5 [file jmir_v23i12e26093_app5.docx]

**Multimedia Appendix 5– Total number of tweets per each topic manually labelled**

| **Topics** | **Label of Dominant Topics** | **Total Tweets (n)** | **Percentage (%)** |
| --- | --- | --- | --- |
| 0 | Emotions | 22,232 | 2.48% |
| 1 | Religion | 19,398 | 2.16% |
| 2 | Duration of Symptoms | 20,948 | 2.34% |
| 3 | Aggravating Factors | 21,119 | 2.35% |
| 4 | Exercise | 19,983 | 2.23% |
| 5 | Dressing | 17,476 | 1.95% |
| 6 | Sleep | 24,639 | 2.75% |
| 7 | Co-Conditions | 18,742 | 2.09% |
| 8 | Sleep | 19,544 | 2.18% |
| 9 | Negative Emotions | 21,883 | 2.44% |
| 10 | Pharmacological | 18,279 | 2.04% |
| 11 | Inactivity | 19,905 | 2.22% |
| 12 | Negative Emotions | 20,826 | 2.32% |
| 13 | Leisure Activities Related To Music | 15,865 | 1.77% |
| 14 | Motherhood | 15,880 | 1.77% |
| 15 | Large Breasts Problem | 15,539 | 1.73% |
| 16 | Pain Regions | 24,247 | 2.70% |
| 17 | Grooming | 15,576 | 1.74% |
| 18 | Positive Experience With Exercise | 17,419 | 1.94% |
| 19 | Self- Treatments | 15,452 | 1.72% |
| 20 | Social Support | 14,816 | 1.65% |
| 21 | Pain Regions | 16,969 | 1.89% |
| 22 | Healthcare Seeking from Health Professional | 14,942 | 1.67% |
| 23 | Female Health Complaints | 16,214 | 1.81% |
| 24 | Daily Life | 14,807 | 1.65% |
| 25 | Lifting | 14,596 | 1.63% |
| 26 | Negative Emotions | 14,489 | 1.62% |
| 27 | Body Positions | 14,173 | 1.58% |
| 28 | Positive Emotions | 12,270 | 1.37% |
| 29 | Work | 13,951 | 1.56% |
| 30 | Symptom Experience | 13,142 | 1.47% |
| 31 | Aggravating Factors | 12,225 | 1.36% |
| 32 | Healthcare | 12,832 | 1.43% |
| 33 | Manual Therapy | 16,766 | 1.87% |
| 34 | Other Causes of Back Pain | 12,305 | 1.37% |
| 35 | Non-Specific Symptomatology | 12,664 | 1.41% |
| 36 | Exercise | 13,478 | 1.50% |
| 37 | Sleep | 10,826 | 1.21% |
| 38 | Studying | 11,798 | 1.32% |
| 39 | Allied Healthcare Food | 16,353 | 1.82% |
| 40 | Food and Drink | 11,551 | 1.29% |
| 41 | Sleep | 14,293 | 1.59% |
| 42 | Pain Regions | 11,688 | 1.30% |
| 43 | Other Symptomatology | 14,291 | 1.59% |
| 44 | Negative Emotions | 12,748 | 1.42% |
| 45 | Pain Regions | 13,962 | 1.56% |
| 46 | Time | 11,454 | 1.28% |
| 47 | Sport | 12,986 | 1.45% |
| 48 | Weather | 10,109 | 1.13% |
| 49 | Office Work and Posture | 13,889 | 1.55% |
| 50 | Negative Emotions | 11,556 | 1.29% |
| 51 | Pain Descriptor | 11,387 | 1.27% |
| 52 | Not Being Understood | 9,180 | 1.02% |
| 53 | Household Cleaning | 11,711 | 1.31% |
| 54 | Negative Emotions | 10,889 | 1.21% |
| 55 | Negative Emotions | 9,775 | 1.09% |
| 56 | Tackling the Day | 9,619 | 1.07% |
| 57 | Entertainment | 10,316 | 1.15% |
| 58 | Catastrophising | 11,396 | 1.27% |
| 59 | Positive Attitude | 9,499 | 1.06% |
