# Supplementary material for: Investigating Individuals’ Perceptions Regarding the Context Around the Low Back Pain Experience: Topic Modeling Analysis of Twitter Data
Source: J Med Internet Res. 2021 Dec 23;23(12):e26093. doi: 10.2196/26093 (PMC8738994; doi:10.2196/26093)
Supplement: Multimedia Appendix 6 [file jmir_v23i12e26093_app6.docx]

**Multimedia Appendix 6 - The 19 contextual categories related to low back pain**

| **Topic No** | **Topic Label** | **High Level Category** |
| --- | --- | --- |
| 3 | Aggravating Factors | Aggravating Factors |
| 31 | Aggravating Factors |  |
| 34 | Other Causes of Back Pain |  |
| 7 | Co-Conditions | Co-Conditions |
| 5 | Dressing | Daily Life |
| 17 | Grooming |  |
| 24 | Daily Life |  |
| 38 | Studying |  |
| 46 | Time |  |
| 56 | Tackling the Day |  |
| 0 | Emotions | Emotion and Beliefs |
| 9 | Negative Emotions |  |
| 12 | Negative Emotions |  |
| 26 | Negative Emotions |  |
| 28 | Positive Emotions |  |
| 44 | Negative Emotions |  |
| 50 | Negative Emotions |  |
| 54 | Negative Emotions |  |
| 55 | Negative Emotions |  |
| 58 | Catastrophising |  |
| 59 | Positive Attitude |  |
| 29 | Work | Employment |
| 49 | Office work And Posture |  |
| 13 | Leisure Activities Related to Music | Entertainment |
| 57 | Entertainment |  |
| 40 | Food and Drink | Food and Drink |
| 22 | Healthcare Seeking from Health Professional | Healthcare |
| 32 | Healthcare |  |
| 33 | Manual Therapy |  |
| 39 | Allied Healthcare Food |  |
| 16 | Pain Regions | Pain Regions |
| 21 | Pain Regions |  |
| 42 | Pain Regions |  |
| 45 | Pain Regions |  |
| 10 | Pharmacological | Pharmacological Therapist |
| 4 | Exercise | Physical Activity |
| 11 | Inactivity |  |
| 18 | Positive Experience with Exercise |  |
| 25 | Lifting |  |
| 27 | Body Positions |  |
| 36 | Exercise |  |
| 47 | Sport |  |
| 53 | Household Cleaning |  |
| 1 | Religion | Religion |
| 19 | Self- Treatments | Self- Treatments |
| 6 | Sleep | Sleep |
| 8 | Sleep |  |
| 37 | Sleep |  |
| 41 | Sleep |  |
| 20 | Social Support | Social Support |
| 2 | Duration of Symptoms | Symptoms |
| 30 | Symptom Experience |  |
| 35 | Non-Specific Symptomatology |  |
| 43 | Other Symptomatology |  |
| 51 | Pain Descriptor |  |
| 48 | Weather | Weather |
| 14 | Motherhood | Women |
| 15 | Large Breasts Problem |  |
| 23 | Female Health Complaints |  |
| 52 | Not Being Understood | Not Being Understood |
