# Supplementary material for: Investigating Individuals’ Perceptions Regarding the Context Around the Low Back Pain Experience: Topic Modeling Analysis of Twitter Data
Source: J Med Internet Res. 2021 Dec 23;23(12):e26093. doi: 10.2196/26093 (PMC8738994; doi:10.2196/26093)
Supplement: Multimedia Appendix 7 [file jmir_v23i12e26093_app7.docx]

**Multimedia Appendix 7 - The total and percentage of tweets for each contextual category**

| **Top Level Factors** | **Total Tweets** | **The Percentage (%)** |
| --- | --- | --- |
| Emotion and Beliefs | 157,563 | 17.57% |
| Physical Activity | 124,251 | 13.85% |
| Daily Life | 80,730 | 9.00% |
| Symptoms | 72,432 | 8.08% |
| Sleep | 69,302 | 7.73% |
| Pain Regions | 66,866 | 7.46% |
| Healthcare | 60,893 | 6.79% |
| Women | 47,633 | 5.31% |
| Aggravating Factors | 45,649 | 5.09% |
| Employment | 27,840 | 3.10% |
| Entertainment | 26,181 | 2.92% |
| Religion | 19,398 | 2.16% |
| Co-Conditions | 18,742 | 2.09% |
| Pharmacological Therapies | 18,279 | 2.04% |
| Self- Treatments | 15,452 | 1.72% |
| Social Support | 14,816 | 1.65% |
| Food and Drink | 11,551 | 1.29% |
| Weather | 10,109 | 1.13% |
| Not Being Understood | 9,180 | 1.02% |
